# Complete genome sequences of classical swine fever virus: Phylogenetic and evolutionary analyses

**DOI:** 10.3389/fmicb.2022.1021734

**Published:** 2022-09-26

**Authors:** Yue Liu, Amina Nawal Bahoussi, Pei-Hua Wang, Changxin Wu, Li Xing

**Affiliations:** ^1^Institute of Biomedical Sciences, Shanxi University, Taiyuan, China; ^2^The Key Laboratory of Medical Molecular Cell Biology of Shanxi Province, Shanxi University, Taiyuan, China; ^3^Shanxi Provincial Key Laboratory for Prevention and Treatment of Major Infectious Diseases, Taiyuan, China

**Keywords:** phylogenetic analysis, recombination, NS5 protein, attenuated vaccines, classical swine fever virus (CSFV)

## Abstract

The classical swine fever virus (CSFV) outbreaks cause colossal losses of pigs and drastic economic impacts. The current phylogenetic CSFV groups were determined mainly based on the partial genome. Herein, 203 complete genomic sequences of CSFVs collected worldwide between 1998 and 2018 available on the GenBank database were retrieved for re-genotyping and recombination analysis. The maximum likelihood phylogenetic tree determined two main groups, GI and GII, with multiple sub-genotypes. The “strain 39” (GenBank ID: AF407339), previously identified as belonging to sub-genotypes 1.1 or 2.2 based on the partial sequences, is found to be genetically distinct and independent, forming a new lineage depicted as GI-2.2b. Ten potential natural recombination events were identified, seven of which were collected in China and found involved in the genetic diversity of CSFVs. Importantly, the vaccine strains and highly virulent strains were all involved in the recombination events, which would induce extra challenges to vaccine development. These findings alarm that attenuated vaccines should be applied with discretion and recommend using subunit vaccines in parallel with other preventive strategies for better management of CSFVs.

## Introduction

Classical swine fever (CSF), caused by CSF virus (CSFV), is a highly contagious disease affecting the *Suidae* family and causing massive pig production losses with severe global economic recession (Moennig, 2000). The clinical manifestations of CSF can range from acute to subacute and chronic forms depending on strain virulence and inducing various symptoms, including hyperthermia, anorexia, depression, vomiting, diarrhea, and skin hemorrhages (Moennig et al., 2003). CSF was first observed in the 1830s in Ohio in the United States of America, and since then, it has been reported globally, mainly in countries of America, Asia, and Eastern Europe (Edwards et al., 2000). In 1978, CSF was successfully eradicated in the USA; in 1963, Canada was free of CSF; the last case recorded in Chile was in 1996; Australia and New Zealand also achieved a CSF-free status in 1962 and 1953, respectively (Edwards et al., 2000). Although eradicated in many countries, CSFV is still endemic in the wild boar population, which causes sporadic outbreaks with an increased risk of re-emergence in domestic pigs, where direct and indirect transmissions have been demonstrated (Moennig, 2015; Xiang et al., 2017; Postel et al., 2019).

CSFV is a small enveloped virus containing a single-stranded, positive-sense RNA genome of approximately 12.3 kb. CSFV belongs to the *Pestivirus* genus in the *Flaviviridae* family (Thiel et al., 1991). The viral genome possesses a single open reading frame (ORF) of approximately 11.7 kb, which is flanked by a 5′-untranslated region (5′-UTR), and a 3′-UTR and encodes a single polyprotein of ~3,898 amino acids (Rümenapf et al., 1991, 1993; Thiel et al., 1991), that is processed during the virus replication into at least 12 functional units, including eight non-structural and four structural proteins. The non-structural protein (NSP) N-terminal protease (N^pro^) is located at the N-terminus of the polyprotein, followed by the four structural proteins, *that is*, the core protein (C), envelope glycoprotein with RNase activity (Erns), and envelope glycoproteins E1 and E2. The remaining seven non-structural proteins (p7, NS2, NS3, NS4A, NS4B, NS5A, and NS5B) are located at the C-terminus of the polyprotein (Thiel et al., 1991; Elbers et al., 1996). E2 glycoprotein and Erns are essential for virus attachment (Blome et al., 2017b). E2 could also form heterodimers with E1 and mediate the receptor binding and the virus entry through endocytosis (Shi et al., 2016).

CSF is a problematic issue for most countries of Asia. In China, CSF was first recognized in the 1920's (Tu et al., 2001), and the highly virulent strain (Shimen) was isolated in 1945 and used as a standard reference for vaccine evaluation. Since then, hundreds of outbreaks have been reported despite the immense efforts to contain the virus (Zhou, 2019). Eradication and elimination of CSFV infection in China, one of the major pig-producing countries, remains a constant challenge (Beer et al., 2015; Fan et al., 2021). Numerous studies revealed changes in the CSFV strains virulence (Shen et al., 2011; Ji et al., 2014; Zhang et al., 2018) and disease pathogenicity from acute to a chronic form, which was suggested to be related to vaccination (Ji et al., 2014).

Vaccination is the major control measure of CSFV in domestic pigs, and live-attenuated vaccines are widely applied in CSFV mandatory control programs in many countries. Those vaccines, including the Russian vaccine strain LK-VNIVViM, the Lapinized Philippines Coronel (LPC) strain, Chinese hog cholera lapinized virus (C-Strain), Japanese guinea-pig exaltation-negative (GPE-) strain, the Mexican PAV strains, and the French cell culture-adapted Thiverval strain, are developed by adaptive mutations after serial passages of main immunogen strains in rabbits or cell culture repeatedly (Baker, 1946; Koprowski et al., 1946; Oláh and Palatka, 1967; Lin et al., 1974; Ji et al., 2014; Coronado et al., 2021). Although live-attenuated vaccines are cost-effective and easy to use, particularly in pregnant sows and young pigs (van Oirschot, 2003), a strong controversy exists, such as in Europe, where domestic pigs are not vaccinated until severe outbreaks occur (Blome et al., 2017a).

CSFV isolates were initially classified into two genotypes based mainly on the complete E2 coding sequences (1119 nt) (Lowings et al., 1996; Postel et al., 2012), which is suggested as the most proper loci for more reliable phylogenetic analysis (Gong et al., 2016; Postel et al., 2019; Izzati et al., 2021; Zhu et al., 2021a). The complete E2 coding sequences are also recommended by the European Union (EU) and Office International des Epizooties (OIE) Reference Laboratory for reliable CSFV phylogenetic analysis (Postel et al., 2012). The global CSFV strains could also be classified into three genotypes and over thirteen subtypes (1.1–1.4, 2.1–2.5, and 3.1–3.4) mainly based on the E2 coding sequence (Zhou, 2019; Izzati et al., 2021; Singh et al., 2022). The correlation between field CSFV virulence and the evolutionary genotypes is not fully understood and not clearly established, although strains belonging to genotype 1 are the most highly virulent, while moderate or low virulent strains belong to genotypes 2 and 3 (Zhu et al., 2021b). Genotype 2 is the most prevalent genotype globally in recent years. Since the 1990's, the CSFV isolates in European countries belong to genotype 2 (2.1, 2.2, or 2.3) (Greiser-Wilke et al., 2000; Biagetti et al., 2001; Blome et al., 2010; Leifer et al., 2010; Postel et al., 2012; Simon et al., 2013) and are genetically distinct from genotype 1. All the American continent CSFV isolates belong to genotype 1. The Argentinian, Brazilian, Columbian, and Mexican isolates form four clusters in subgroup 1.1; the Honduran and Guatemalan CSFV strains are clustered in subgroup 1.3 (Zhou, 2019), and the Cuban isolates form a subgroup 1.4 (Postel et al., 2013). The CSFVs isolated in South Africa CSF outbreak in 2005 and in Israel in 2009 belong to subgroup 2.1 (Zhou, 2019). Several sub-genotypes 1.1, 2.1, 2.2, and 2.4 of CSFV isolates are reported in India, with 1.1 being dominant (Singh et al., 2022).

Since current phylogenetic classifications of CSFV are based mainly on partial genome sequences and may not provide sufficient information that helps fully understand the evolutionary character and genetic relatedness of the circulating strains, we reviewed in this report the full-length genomes of CSFVs by phylogenetical and recombination analysis.

## Re-genotyping of CSFV based on the complete genome sequences

A total of 203 complete genomic sequences of CSFVs, collected worldwide between 1977 and 2018 from 20 countries in Asia, Europe, and America, including China (66), South Korea (52), Japan (2), Mongolia (1), Vietnam (4), India (15), Germany (35), Lithuania (1), Denmark (6), Netherlands (2), Croatia (1), Bulgaria (1), Serbia (2), Spain (1), Russia (1), Sweden (1), Switzerland (7), France (1), USA (3), and Cuba (1), were retrieved from GenBank database. The maximum likelihood phylogenetic tree was constructed using MEGA-X software (Tamura and Nei, 1993; Tamura et al., 2021). The viruses in the current study were identified by their GenBank ID, name, country, and year of collection.

**Figure 1:**
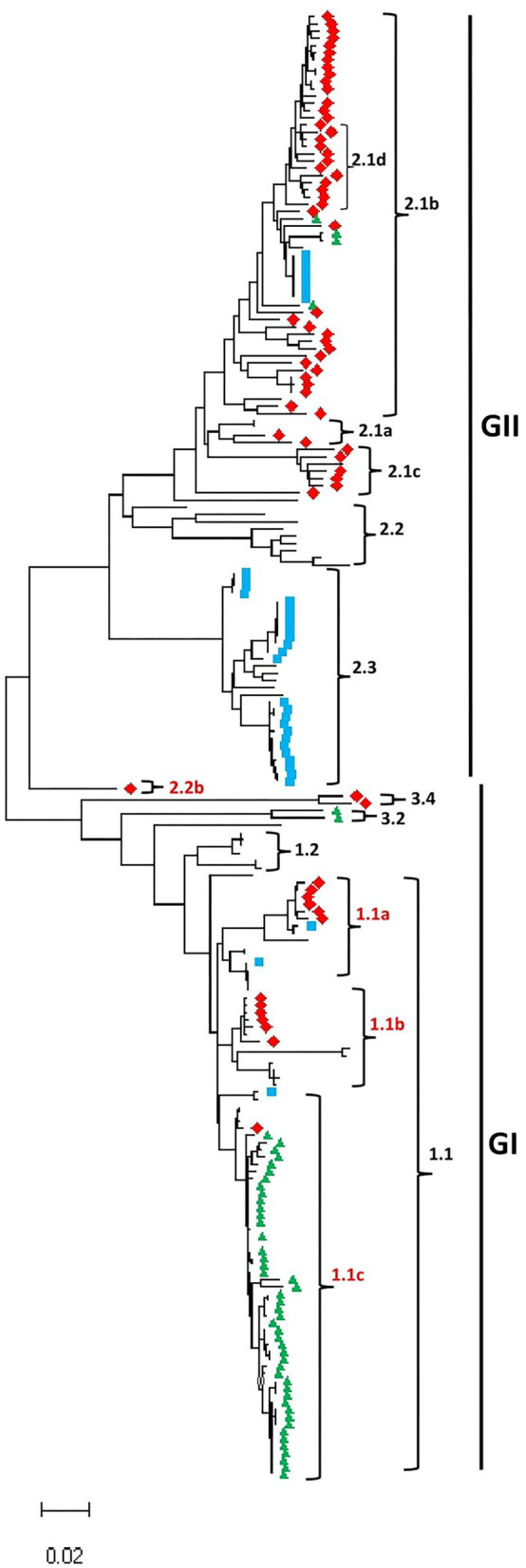
Maximum likelihood phylogenetic tree based on the full-length genome of CSFV isolated worldwide from 1998 to 2018 (continue in Supplementary Figures 1–3). Multiple-sequence alignments were performed using Clustal Omega server, and the phylogenetic tree was constructed from the aligned nucleotide sequences using the maximum likelihood method in the MEGA-X software. The numbers at each branch represent bootstrap values of 1,000 replicates. The scale bars indicate the number of inferred substitutions per site. Viruses isolated in China are indicated with red diamonds, South Korea in green triangles, and Germany in blue squares.

As shown in Figure 1, Supplementary Table 1, and Supplementary Figures 1–3, the CSFV full-length genome sequences cluster into two main groups, GI and GII. The GI group includes 95 CSFV strains and contains five sub-genotypes: 1.1, 1.2, 2.2b, 3.2, and 3.4; meanwhile GII group includes 108 CSFV strains and contains five sub-genotypes: 2.1a, 2.1b, 2.1c, 2.2, and 2.3. We identified a mixed population of CSFV genotypes and sub-genotypes co-circulating in China, Germany, and South Korea, where the earliest strains fall into GI while the more recently collected strains fall into GII except South Korea CSFV strains. Interestingly, the GI-2.2b is a new sub-genotype identified based on the full-length genome phylogenetic analysis and is found restricted to China. These findings corroborate the previous genotyping based on the partial genomic sequences, such as 5′-UTR and E2 gene et al., where most of the strains included in this study fall into their corresponding sub-genotypes. For example, the strain CSF1048 (GenBank ID: HQ148063) assigned in 2.1b based on the complete E2 genomic sequences (Leifer et al., 2011) clusters into 2.1b based on the full-length genome and is genetically close to China strains GXWZ02 (GenBank ID: AY367767) (Li et al., 2006), SXYL2006 (GenBank ID: GQ122383) (Li et al., 2011), Zj0801 (GenBank ID: FJ529205), HEBZ (GenBank ID: GU592790), and 0406/CH (GenBank ID: AY568569). Similarly, Paderborn (GenBank ID: AY072924), assigned to 2.1a based on the partial E2 sequences (Uttenthal et al., 2001), clusters in 2.1a in the GII based on the full-length genome and is genetically close to two China strains SXCDK (GenBank ID: GQ923951) and 96TD (GenBank ID: AY554397). Viruses HNLY-2011 (GenBank ID: JX262391) and HNSD-2012 (GenBank ID: JX218094) were reported as a new sub-genotype 2.1c (Jiang et al., 2013) and genetically close to the virus HY78 (GenBank ID: MH979231.1) isolated in Vietnam in 2015 (Kim et al., 2019). The first identified sub-genotype 2.2 virus LAL-290 (GenBank ID: KC851953) (Kumar et al., 2014) still forms an independent lineage 2.2 in group GII with other genetically closer genomes. The reference strain Alfort/Tuebingen (GenBank ID: J04358) assigned to 2.3 based on 5′-UTR, or partial E2 gene (Meyers et al., 1989), and Uelzen (GenBank ID: GU324242), Euskirchen (GenBank ID: GU233732), Borken (GenBank ID: GU233731), Roesrath (GenBank ID: GU233734), and Hennef (GenBank ID: GU233733) assigned to 2.3 based on complete genome, 5′-UTR, N^pro^, or E2 gene (Leifer et al., 2010) cluster into the same sub-genotype 2.3 in this study. The highly virulent strain Brescia (GenBank ID: M31768), assigned to 1.2 based on 5′-UTR, or partial E2 gene (Moormann et al., 1990), fall into 1.2 based on the full-length genome sequences and is genetically close to the attenuated vaccine strain RUCSFPLUM (GenBank ID: AY578688) isolated in 2001 (Risatti et al., 2005).

CSFV strains, P97 (GenBank ID: L49347) (Shiu et al., 1996) and TW-94 (GenBank ID: AY646427), were reported as a new sub-genotype 3.4 based on the complete genome sequences (Lin et al., 2007); YI9908 (GenBank ID: KT716271) and JJ9811 (GenBank ID: KF669877.1) isolated in South Korea form an independent sub-genotype 3.2 (Lim et al., 2016). Both 3.4 and 3.2 fall into the GI group, genetically closer to 1.2 and 1.1. The sub-genotype 1.1 could be further divided into 1.1a, 1.1b, and 1.1c based on the full-genome sequences in this analysis. The highly virulent strain Koslov (GenBank ID: HM237795), previously assigned as 1.1 based on complete genome, 5′-NTR, N^pro^, or E2 protein sequences (Leifer et al., 2010), clusters into 1.1a with the strain HCLV (GenBank ID: AF091507). The NG79-11 (GenBank ID: KC503764) (Tomar et al., 2015) and VB-131 (GenBank ID: KM262189) (Kamboj et al., 2014) isolated in India are assigned as 1.1b together with Shimen strain (GenBank ID: AF092448), a highly virulent strain isolated in China in 1945 (Zhang et al., 2018), SWH (GenBank ID: DQ127910) (Li et al., 2006), and GZ-2009 (GenBank ID: HQ380231) (Shen et al., 2011). Alfort/187 (GenBank ID: X87939) (Ruggli et al., 1996) was under 1.1c together with the high virulent strains Glentorf (GenBank ID: U45478), CAP (GenBank ID: X96550), and Alfort A19 (GenBank ID: U90951), and the most of viruses isolated in South Korea during 1987–2019.

Compared to partial genomic sequence-based analysis, the complete genome sequence-based phylogenetic analysis would provide a more accurate understanding of the genetic relatedness of CSFV “Strain 39” (GenBank ID: AF407339) belonging to sub-genotypes 1.1 or 2.2 based on the 5'-UTR fragment or the entire 5'-UTR-E2 sequences, respectively (Postel et al., 2012), was recently indicated to be genetically closer to the Indian strain CSFV IND/UK/LAL-290 (GenBank ID: KC851953) in 2.2 based on complete E2 sequences (Zhu et al., 2021b). Herein, the full-length genomic sequence revealed that “Strain 39” is genetically distinct from viruses of 1.1 or 2.2 sub-genotypes and forms a new independent lineage depicted as GI-2.2b in this analysis.

A group of viruses including JSZL (GenBank ID: KT119352), SDSG1410 (GenBank ID: MF150645), SDLS1410 (GenBank ID: MF150644) (Zhang et al., 2015), HB150309 (GenBank ID: MF150640), JL150418 (GenBank ID: MF150642), NK150425 (GenBank ID: MF150643), SDZC150601 (GenBank ID: MF150646), HLJ1 (GenBank ID: MF150641), and SDWF-2016 (GenBank ID: MK211486) (Zhang et al., 2018) were claimed as a new sub-genotype 2.1d based on full or partial E2 sequences. However, according to Figure 1, these 2.1d sub-genotype isolates are genetically within the 2.1b sub-genotype and do not form a distinct lineage as reported before. As shown in Figure 1 and Supplementary Table 1, the China strains could be found in most of sub-genotypes.

To increase the stringency and reliability of our phylogenetic tree findings, we performed a similarity analysis, comparing the complete genome of HEBZ (GenBank ID: GU592790) in 2.1b sub-genotype to 13 representative CSFV full-length sequences for each sub-genotypes using SimPlot analysis (Lole et al., 1999). As shown in Supplementary Figure 4, the E1, E2, NS2, NS4, and NS5 coding regions revealed low similarity levels, where strains from GI exhibited the lowest percentage levels (<80%) and are shown distant from those of GII that exhibited higher similarity percentage (>90%). However, the same genomic fragments displayed distinct similarity levels among strains of GII group ranging between 90 and 98%, contrary to strains of GI group (Supplementary Figure 4). The latter indicates the divergences between CSFV sub-genotypes and corroborates the high diversity of GII group (Figure 1). Interestingly, “strain 39” (GenBank ID: AF407339), which formed a new lineage 2.2b in GI, resembled highly GII before NS4B coding region, but become closer to GI in NS5 coding region. These findings are consistent with the phylogenetic analysis results, suggesting that the defined CSFV genotypes and sub-genotypes are distinctly shown and highly specific, with a significant difference in the genome between GI and GII (Supplementary Figure 4).

## Genomic recombination between the worldwide collected CSFVs

**Figure 2:**
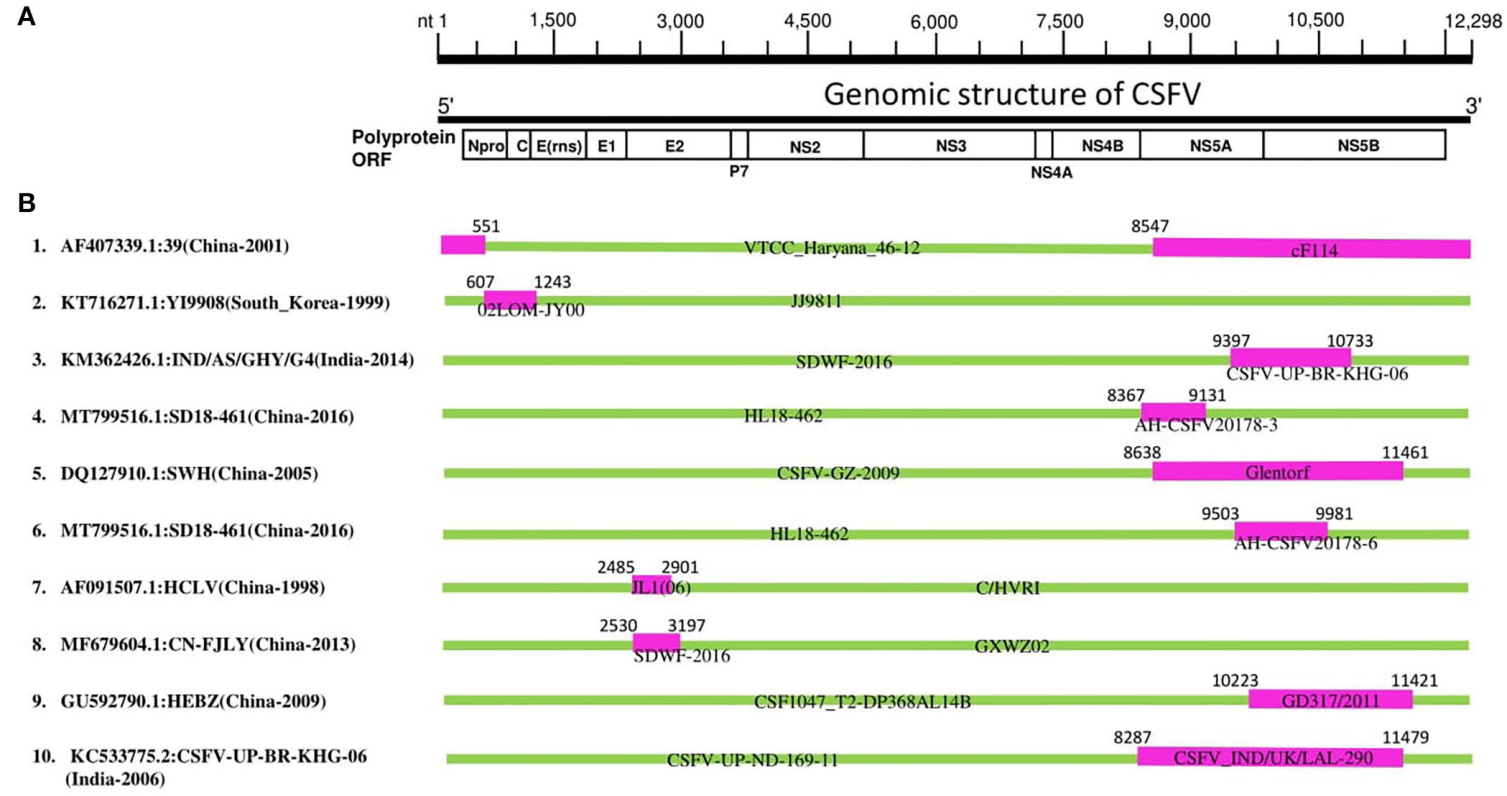
CSFV complete genome recombination. **(A)** Diagram showing the full-length genome of CSFV strain GD 19/2011 (GenBank ID: KU 504339) and the corresponding regions encoding N^pro^ C, Erns, E1, E2, p7 N2, NS3, NS4A, NS4B, NS5A, and NS5B. **(B)** Schematic representation of potential recombination events listed in Supplementary Table 2. The recombination event serial number and the description of potential recombinants (GenBank ID: virus name/country-collection year) are shown on the left. The filled pink and green blocks represent the genomic regions from minor or major parent viruses, respectively. The numbers on the top of filled green blocks indicate the nucleotide positions of breakpoints on the corresponding recombinant viruses.

To understand the mechanisms behind the adaptation and genomic diversity of the worldwide circulating CSFVs, we conducted a recombination analysis of 203 complete genomic sequences of CSFV using the seven algorithms of the RDP4 software package (Martin et al., 2015). Our recombination analysis identified 10 natural recombination events (Supplementary Table 2), six of which occurred between GII strains, three occurred between GI strains, and only one was intergenotype (GI vs GII) (Supplementary Table 2, Figure 2). Importantly, most of the identified recombinant strains were collected in China (Events 1, 4, 5, 6, 7, 8, and 9), four events among which occurred between China strains (Events 4, 6, 7, and 8), two between China and Germany strains (Events 5 and 9), and one occurred between China and India strains (Event 1) (Supplementary Table 2, Figure 2). Furthermore, recombination occurred between South Korean strains (Event 2) and between Indian strains (Event 10) (Supplementary Table 2, Figure 2).

In previous studies (He et al., 2007; Chen and Chen, 2014), the “strain 39” (GenBank ID: AF407339) isolated in China has been identified to stem from nature recombination. Consistently, “strain 39” in our report is identified as a recombinant strain (Event 1) resulting from natural recombination between cF114 (GenBank ID: AF333000.1) and VTCC_Haryana_46-12 (GenBank ID: MK405702) as minor and major parental sequences, respectively, which is supported by similarity analysis where the genome of “strain 39” highly resembled GII before NS4B region, but become closer to GI in NS5 region (Supplementary Figure 4), and also the recombination map where the beginning breakpoint was located at nt 8547 corresponding to NS5 fragment (Figure 2).

Furthermore, Lim et al. reported that the YI9908 strain shares with the JJ9811 strain a 95.7% homology at the nucleotide (nt) level and 95.6% at the amino acid level (Lim et al., 2016). Comparative analysis of YI9908 and JJ9811 strains revealed a low nucleotide sequence homology for the N^pro^ gene (90.1%) and the C gene (87.5%) (Lim et al., 2016). Consistently, our analysis identified YI9908 (GenBank ID: KT716271) as a recombinant strain (Event 2) and the JJ9811 strain (GenBank ID: KF669877) as its major parent (Supplementary Table 2), and the recombination map exhibited the beginning and ending breakpoints at nt 607-1243, corresponding to the N^pro^ and the C genes (Supplementary Figure 5).

It has been reported recently that Chinese CSFV HCLV (AF091507.1) resulted from natural recombination between Shimen (AF092448.2) and CSFV strain C/HVRI (AY805221.1) with two recombination breakpoints at nt 2484 and 2900. Similarly, in our report, HCLV (AF091507.1) is identified as a recombinant strain (Event 7) with identical breakpoints location at nt 2485-2901; however, HCLV is determined resulting from a different parental strain: JL1(06) (EU497410.1) with C/HVRI (AY805221.1). These findings suggest genetic mosaicism of the HCLV virus (Supplementary Table 2, Figure 2).

Importantly, the highly virulent and the vaccine strains are all identified as involved in recombination during the genetic evolution of CSFVs. Our phylogenetic tree revealed that recombinant YI9908 (Event 2) and its major parent JJ9811 are genetically close to the vaccine strains: LK-VNIVViM and Rovac, while its minor parent 02LOM-JY00 is itself a vaccine strain applied in South Korea (Supplementary Figure 3). The recombinant HCLV (Event 7) and its major parent C/HVRI are all vaccine strains used in China, whereas the minor parent JL1(06) (GenBank ID: EU497410) is a highly virulent strain (Supplementary Figure 3). Therefore, the high rate of recombination, identified between China CSFV strains, particularly in the GII-2 group, is a significant threat to the pork industry and might be related to the applied vaccination programs, which should be merited particular attention.

As shown in Figure 2, in eight out of ten CSFV recombination events (Asia strains), the beginning and ending breakpoints are found to be within 5'- or 3'- proximal regions of the genome, encoding the N^pro^, C, NS5A, and NS5B proteins, respectively. The NS5A protein has been demonstrated to regulate the CSFV replication and viral RNA synthesis by interacting with NS5B and 3′-UTR, where low levels of NS5A stimulate the virus replication while high levels of NS5A suppress the RNA replication (Xu et al., 2020). Therefore, these recombination characteristics partly explain the adaptation and evolution of the circulating CSFV strains and predict future severe outbreaks that might challenge vaccine development, where subunit vaccines are highly recommended instead of attenuated forms.

We further verified the authenticity of the identified recombination events by building phylogenetic trees based on different genomic fragments of CSFV (relative to the strain GD19/2011, GenBank: KU504339.1) (Supplementary Figure 5): The fragment nt 6000-7000 encodes NS3 protein, while nt 9500-10000 encodes part of NS5A and NS5B proteins. As shown in Supplementary Figure 5, the phylogenetic trees based on two genome fragments were not superimposed. Notably, the recombinant strains (indicated in red color) of each event are genetically closer to their minor parents (indicated in blue color) in the phylogenetic tree based on nt 9,500–10,000 (Supplementary Figure 5) and become closer to their major parents (indicated with yellow color) in the phylogenetic tree based on nt 6,000-7,000 (Supplementary Figure 5). These results are congruent with the recombination mapping, indicating that the identified recombination events are real.

Therefore, this report exhibited that natural recombination was driving the genetic diversity and complexity of circulating CSFV strains. As the attenuated vaccines were found involved in the recombination events, their application should be cautious, and subunit vaccines are highly recommended for more successful control and effective prevention.

## Data availability statement

The original contributions presented in the study are included in the article/Supplementary material, further inquiries can be directed to the corresponding author.

## Author contributions

AB, YL, and LX conceived the study and revised the manuscript. AB, YL, and P-HW performed analysis. YL and AB wrote the manuscript. LX and CW supervised analysis. All authors read and approved the final manuscript.

## Funding

This work is funded by the Programme of Introducing Talents of Discipline to Universities (D21004) and the Fundamental Research Program of Shanxi Province, China (20210302124187).

## Conflict of interest

The authors declare that the research was conducted in the absence of any commercial or financial relationships that could be construed as a potential conflict of interest.

## Publisher's note

All claims expressed in this article are solely those of the authors and do not necessarily represent those of their affiliated organizations, or those of the publisher, the editors and the reviewers. Any product that may be evaluated in this article, or claim that may be made by its manufacturer, is not guaranteed or endorsed by the publisher.
