# Supplementary material for: Complete genome sequences of classical swine fever virus: Phylogenetic and evolutionary analyses
Source: Front Microbiol. 2022 Sep 26;13:1021734. doi: 10.3389/fmicb.2022.1021734 (PMC9549409; doi:10.3389/fmicb.2022.1021734)
Supplement: Supplementary file 1 [file Data_Sheet_1.PDF]

Supplementary Figures S1-S5

Complete genome sequences of Classical Swine Fever Virus: Phylogenetic and Evolutionary analyses

Yue Liu[1,2,3#], Amina Nawal Bahoussi[1#], Pei-Hua Wang[1], Changxin Wu[1,2,3], Li Xing[1,2,3]*

Supplementary Figures S1, S2, and S3. The maximum likelihood phylogenetic tree based on the full-length genome of CSFV isolated worldwide from 1998 to 2018. Multiple-sequence alignments were performed using Clustal Omega server, and the phylogenetic tree was constructed from the aligned nucleotide sequences using the maximum likelihood method in the MEGA-X software. The numbers at each branch represent bootstrap values of 1000 replicates. The scale bars indicate the number of inferred substitutions per site. Viruses isolated in China are indicated with red diamonds; South Korea in green triangles; Germany in blue triangles.

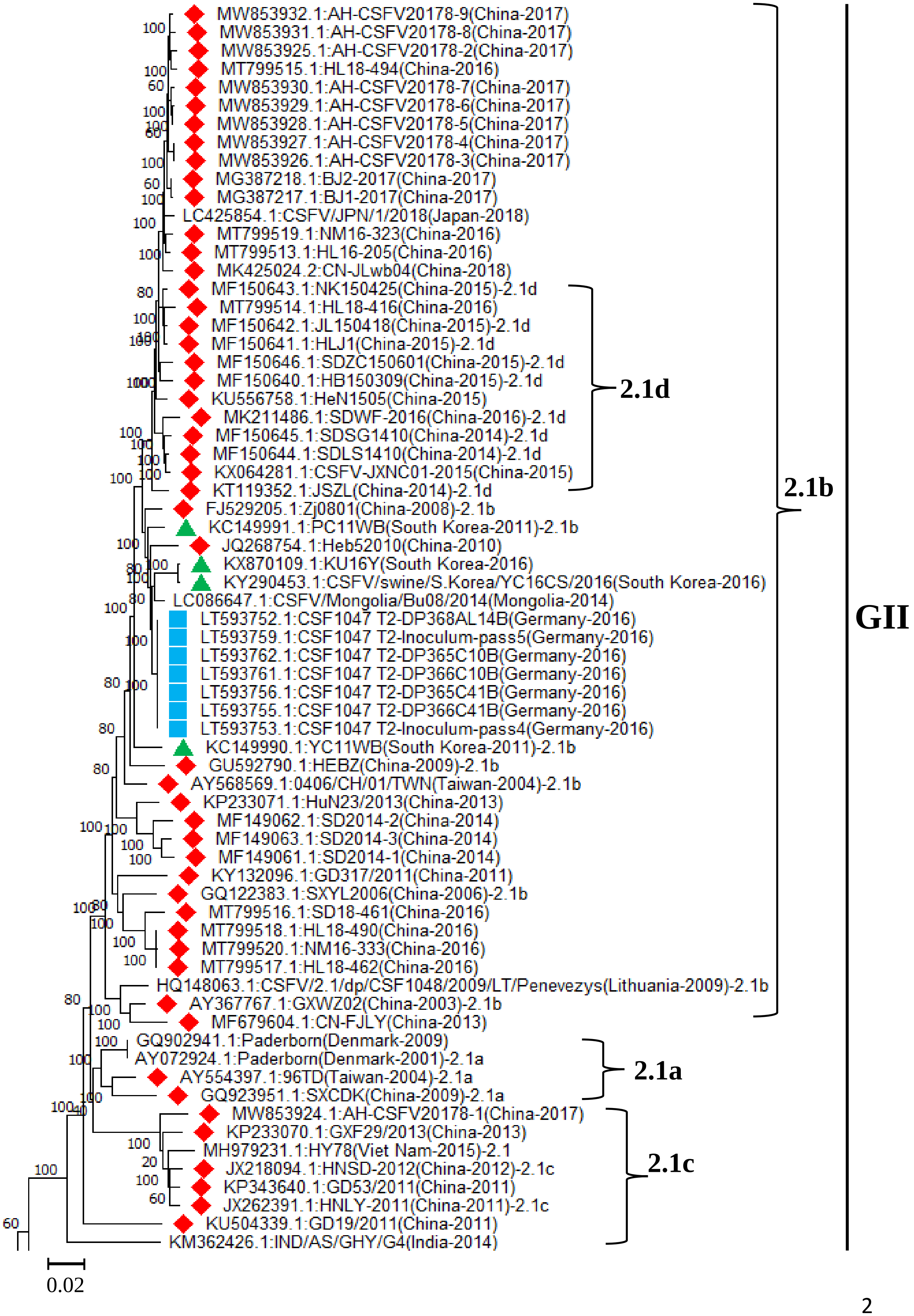

Supplementary Figure S1

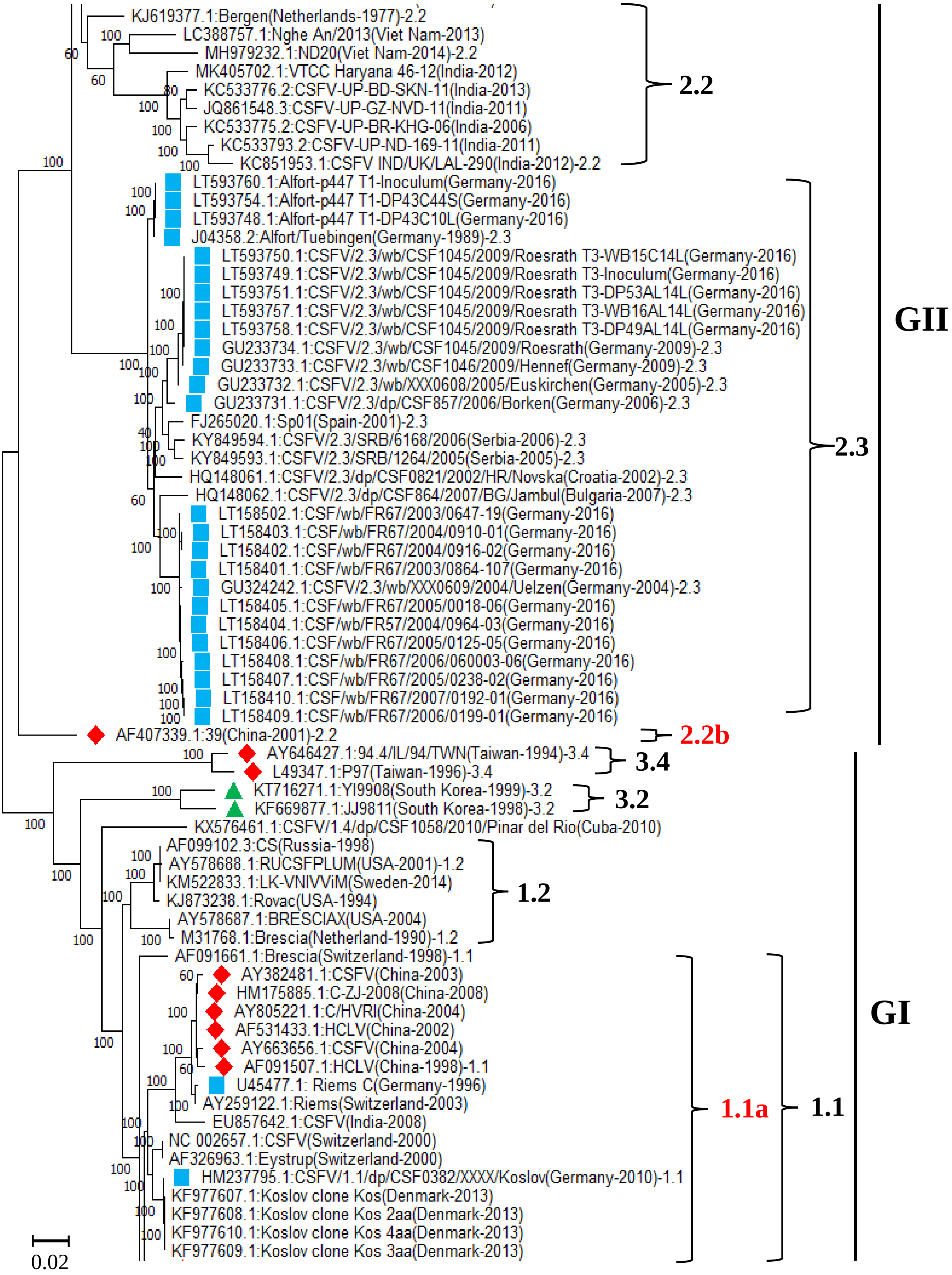

Supplementary Figure S2

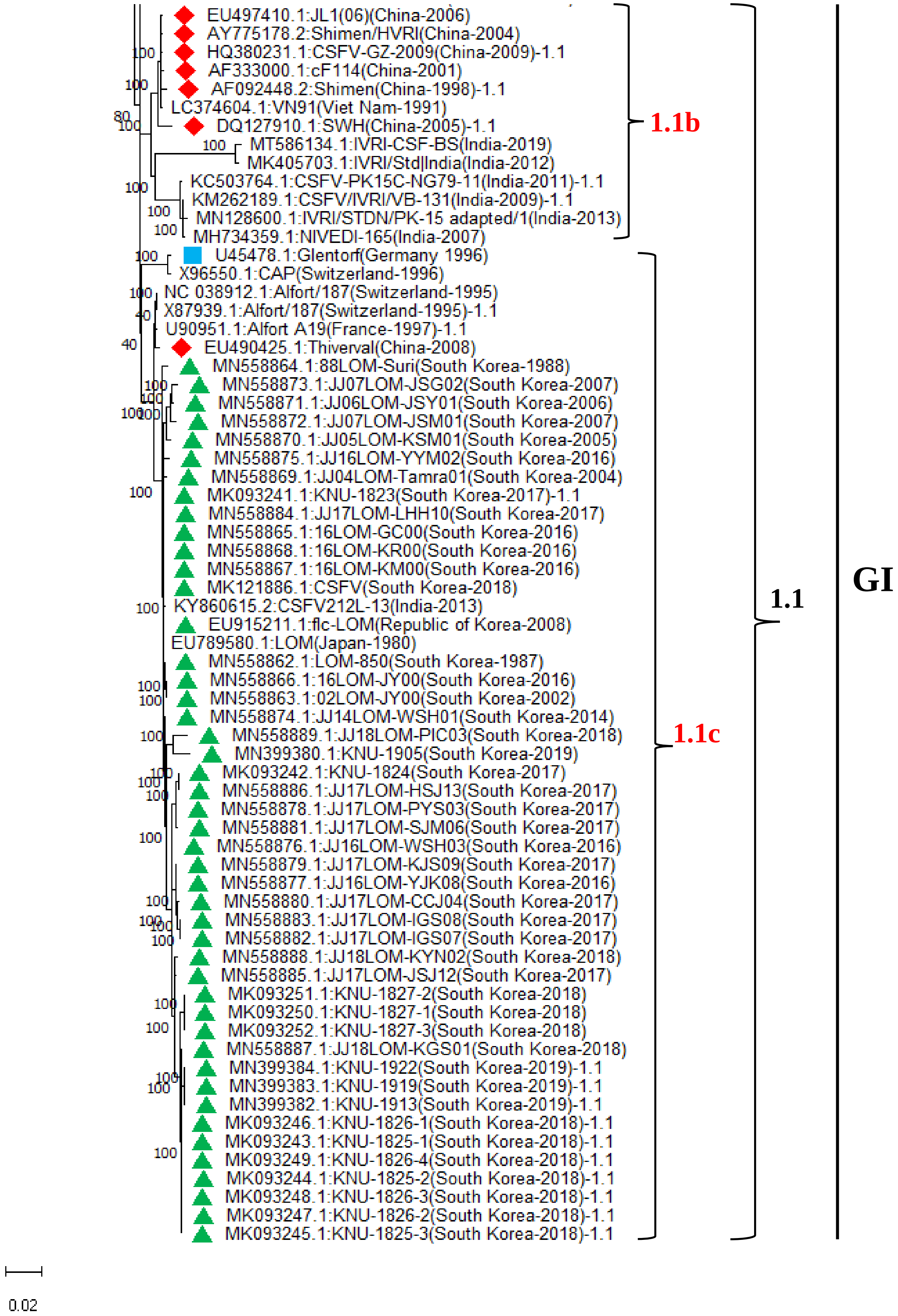

Supplementary Figure S3

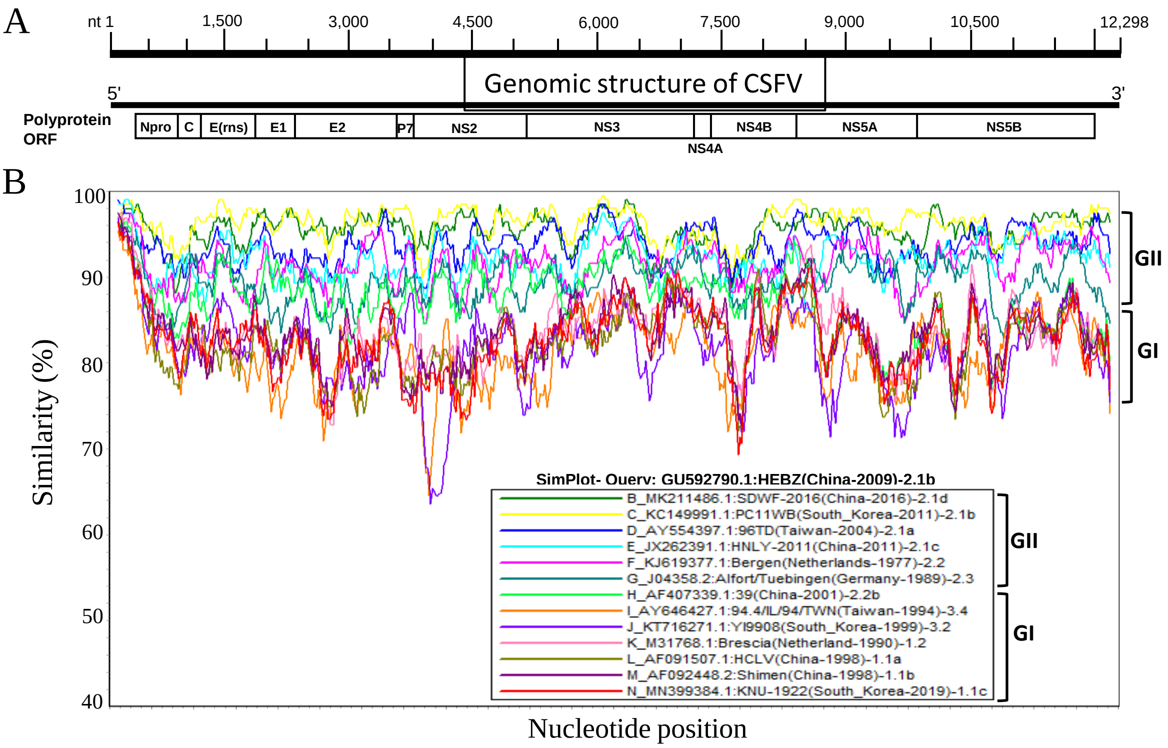

Supplementary Figure S4. Sequence similarity was compared between representative CSFV strains from each sub-genotypes isolated in China. (A). Diagram showing the full-length genome of CSFV strain GD19/2011 (GenBank ID: KU504339) and the corresponding regions encoding N^{pro}, C, Erns, E1, E2, p7, NS2, NS3, NS4A, NS4B, NS5A, and NS5B. (B). Sequence similarity was performed using HL18-494 (GenBank ID: MT799515) as the query.

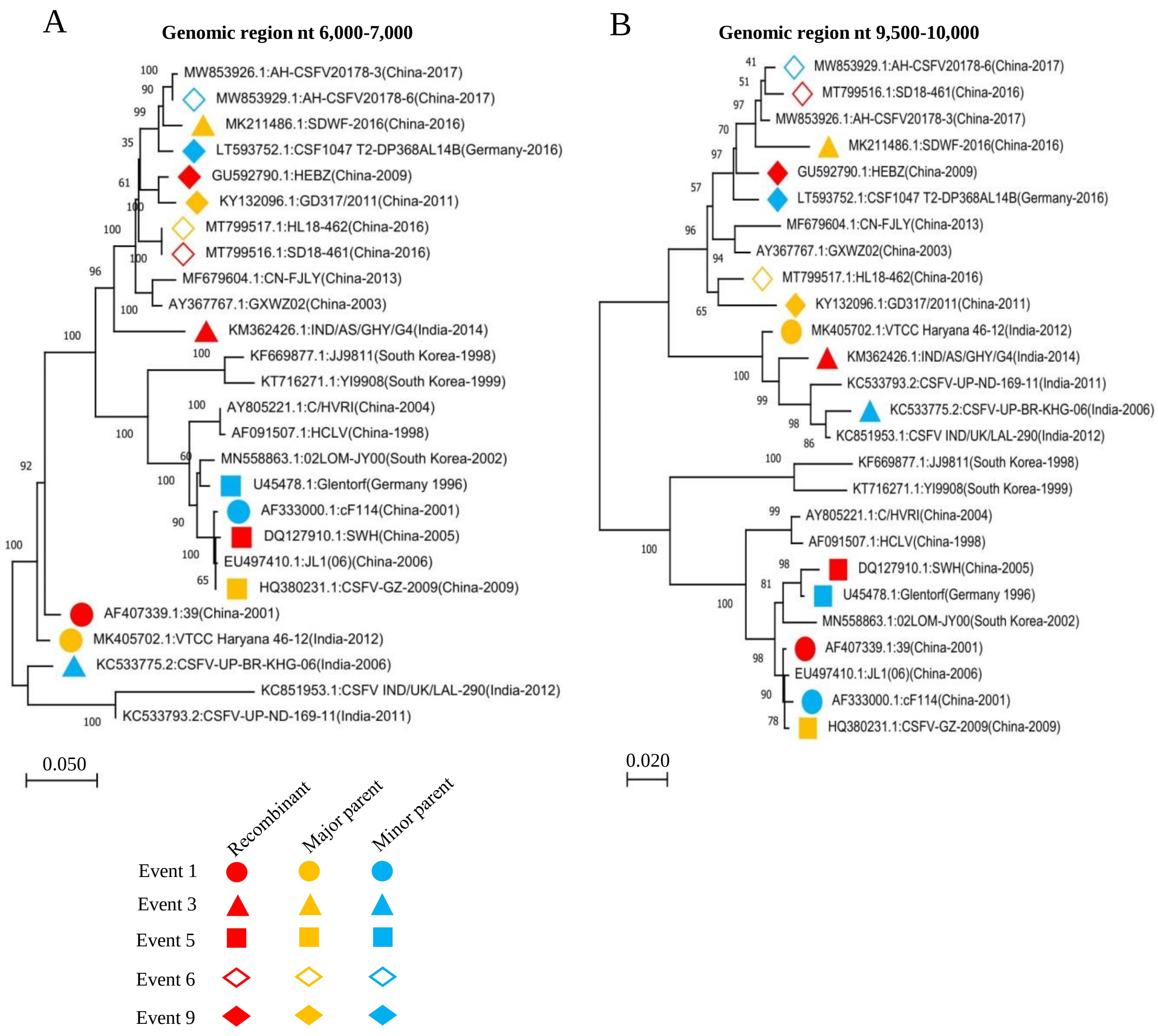

Supplementary Figure S5. The phylogenetic trees based on the indicated genomic fragments of CSFV involved in recombination. The viruses involved in recombination events 1, 3, 5, 6, and 9 were labelled in the phylogenetic trees. The red colour indicates recombinant. The yellow and blue colours indicate major and minor parents, respectively.
