# Supplementary material for: Complete genome sequences of classical swine fever virus: Phylogenetic and evolutionary analyses
Source: Front Microbiol. 2022 Sep 26;13:1021734. doi: 10.3389/fmicb.2022.1021734 (PMC9549409; doi:10.3389/fmicb.2022.1021734)
Supplement: Supplementary file 2 [file Data_Sheet_2.PDF]

Supplementary Table S1.

Complete genome sequences of Classical Swine Fever Virus: Phylogenetic and Evolutionary analyses

Yue Liu[1,2,3#], Amina Nawal Bahoussi[1#], Pei-Hua Wang[1], Changxin Wu[1,2,3], Li Xing[1,2,3]*

Supplementary Table S1. Geographic distribution of CSFV full-length genome-based genotypes.

**Genotype**		**Asia (China)**	**Asia (other countries)**	**Europe**	**America**
GII	2.1b	China	Japan, South Korea, Mongolia	Germany, Lithuania	
	2.1a	China		Denmark	
	2.1c	China	India, Vietnam		
	2.2		India, Vietnam	Netherlands	
	2.3			Croatia, Germany, Bulgaria, Serbia, Spain	
GI	2.2b	China			
	3.4	China (Taiwan)			
	3.2		South Korea		
	1.2			Russia, Sweden, Switzerland	USA, Cuba
	1.1	China	India, Japan, Vietnam, South Korea	Germany, Switzerland, Denmark, France	

Supplementary Table S2. Identification of 10 potential recombination events in the complete genome of CSFVs isolated during 1977 -2018. The potential recombination events were identified by each of 7 algorithms (RDP, GENECONV, Bootscan, MaxChi, Chimaera, SiScan, and 3Seq) embedded in the RDP4 package. Genogroup was defined based on the phylogenetic tree of CSFV full-length genome in this study (see Figure 1).

**Event serial number**	**Recombinant**		**Minor parent**		**Major parent**		**Detection methods**						
	GenBank ID: Virus name （Country-Year）	Genotype	GenBank ID: Virus name （Country-Year）	Genotype	GenBank ID: Virus name （Country-Year）	Genotype	R	G	B	M	C	S	T
1	AF407339.1:39(China-2001)	**GI-2.2c**	AF333000.1:cF114(China-2001)	**GI-1.1**	MK405702.1:VTCC_Haryana_46-12(India-2012)	**GII-2.2**	+	+	+	+	+	+	+
2	KT716271.1:YI9908(South_Korea-1999)	**GI-3.2**	MN558863.1:02LOM-JY00(South_Korea-2002)	**GI-1.1b**	KF669877.1:JJ9811(South_Korea-1998)	**GI-3.2**	+	+	+	+	+	+	+
3	KM362426.1:IND/AS/GHY/G4(India-2014)	**GII-2.1c**	KC533775.2:CSFV-UP-BR-KHG-06(India-2006)	**GII-2.2**	MK211486.1:SDWF-2016(China-2016)	**GII-2.1b**	+	+	+	+	+	+	+
4	MT799516.1:SD18-461(China-2016)	**GII-2.1b**	MW853926.1:AH-CSFV20178-3(China-2017)	**GII-2.1b**	MT799517.1:HL18-462(China-2016)	**GII-2.1b**	+	+	+	+	+	+	+
5	DQ127910.1:SWH(China-2005)	**GI-1.1**	U45478.1:Glentorf(Germany_1996)	**GI-1.1**	HQ380231.1:CSFV-GZ-2009(China-2009)	**GI-1.1**	+	+	+	+	+	+	+
6	MT799516.1:SD18-461(China-2016)	**GII-2.1b**	MW853929.1:AH-CSFV20178-6(China-2017)	**GII-2.1b**	MT799517.1:HL18-462(China-2016)	**GII-2.1b**	+	+	+	+	+	+	+
7	AF091507.1:HCLV(China-1998)	**GI-1.1**	EU497410.1:JL1(06)(China-2006)	**GI-1.1**	AY805221.1:C/HVRI(China-2004)	**GI-1.1**	+	+	+	-	+	-	+
8	MF679604.1:CN-FJLY(China-2013)	**GII-2.1b**	MK211486.1:SDWF-2016(China-2016)	**GII-2.1b**	AY367767.1:GXWZ02(China-2003)	**GII-2.1b**	+	+	+	-	+	-	+
9	GU592790.1:HEBZ(China-2009)	**GII-2.1b**	LT593752.1:CSF1047_T2-DP368AL14B(Germany-2016)	**GII-2.1b**	KY132096.1:GD317/2011(China-2011)	**GII-2.1b**	+	+	+	+	+	+	+
10	*KC533775.2:CSFV-UP-BR-KHG-06(India-2006)	**GII-2.2**	KC851953.1:CSFV_IND/UK/LAL-290(India-2012)	**GII-2.2**	KC533793.2:CSFV-UP-ND-169-11(India-2011)	**GII-2.2**	+	+	+	+	+	-	+

R, RDP; G, GENECONV; B, BootScan; M, MaxChi; C, Chimaera; S, SiScan; T, 3Seq.

* The major or minor parent may be the actual recombinant due to the possibility of misidentification.
